# Preservation of Fluorescence Signal and Imaging Optimization for Integrated Light and Electron Microscopy

**DOI:** 10.3389/fcell.2021.737621

**Published:** 2021-12-15

**Authors:** Pieter Baatsen, Sergio Gabarre, Katlijn Vints, Rosanne Wouters, Dorien Vandael, Rose Goodchild, Sebastian Munck, Natalia V. Gounko

**Affiliations:** ^1^ VIB-KU Leuven Center for Brain and Disease Research, Electron Microscopy Platform and VIB-Bioimaging Core, Leuven, Belgium; ^2^ KU Leuven Department of Neurosciences, Leuven Brain Institute, Leuven, Belgium; ^3^ VIB-KU Leuven Center for Brain and Disease Research, Light Microscopy Expertise Unit and VIB Bioimaging Core, Leuven, Belgium; ^4^ VIB-KU Leuven Center for Brain and Disease Research, Laboratory for Membrane Trafficking, Leuven, Belgium; ^5^ VIB-KU Leuven Center for Brain and Disease Research, Laboratory for Dystonia Research, Leuven, Belgium

**Keywords:** in-resin-fluorescence, correlated light and electron microscope, integrated light and electron microscope, electron microscopy, light microscopy

## Abstract

Life science research often needs to define where molecules are located within the complex environment of a cell or tissue. Genetically encoded fluorescent proteins and or fluorescence affinity-labeling are the go-to methods. Although recent fluorescent microscopy methods can provide localization of fluorescent molecules with relatively high resolution, an ultrastructural context is missing. This is solved by imaging a region of interest with correlative light and electron microscopy (CLEM). We have adopted a protocol that preserves both genetically-encoded and antibody-derived fluorescent signals in resin-embedded cell and tissue samples and provides high-resolution electron microscopy imaging of the same thin section. This method is particularly suitable for dedicated CLEM instruments that combine fluorescence and electron microscopy optics. In addition, we optimized scanning EM imaging parameters for samples of varying thicknesses. These protocols will enable rapid acquisition of CLEM information from samples and can be adapted for three-dimensional EM.

## Introduction

Our ability to define the molecular and cellular mechanisms of life often requires that specific molecules are detected in cell or tissue samples in the context of various subcellular structures. Correlative light and electron microscopy (CLEM) does so by marking target molecules in a sample in such a way that they can be visualized by light microscopy (LM), and their cellular context studied by electron microscopy (EM). CLEM has been practiced in a more rudimentary form for many years, employing enzyme-based (e.g., horseradish peroxidase) markers and specific stains, and using mainly transmission electron microscopy (TEM) of osmicated thin sections. However, CLEM has evolved over the last decade to a more mature methodology with different approaches, specialized equipment and employing both scanning electron microscopy (SEM) and TEM (Müller-Reichert and Verkade, 2012). Currently, the most frequent way of marking molecules of interest is by using fluorescent tags and then correlating fluorescence with ultrastructural EM imaging of the same sample. Of particular interest are approaches that image living cells using fluorescence microscopy (FM) and then correlate these data with EM images acquired after fixation, high-pressure freezing, and freeze-substitution (Brown et al., 2009). Alternatively, fluorescence in a region of interest can be imaged, outlined by laser branding, and the laser marks re-identified in EM for post-hoc co-registration of the two images (Urwyler et al., 2014; Vints et al., 2021). However, these methods are time-consuming and are often comparatively imprecise. The problem of inaccurate correlation represents a major issue for understanding the relationship between individual molecules and small subcellular structures, for example, intracellular vesicles, organelle contact sites, and small subcellular domains such as the neuronal synapse.

A fundamental issue in achieving high accuracy CLEM is that most FM samples are hundreds of nanometers or micrometers thick, while TEM requires ultrathin sections (50–100 nm). Therefore, the best way to alleviate the Z-integration problem is to simultaneously image the same thin section with both types of microscopy. New specialized CLEM instruments have been developed that combine FM and EM optics in one unit to facilitate this approach (Agronskaia et al., 2008; Peddie et al., 2014). While one type of integrated light and electron microscope (ILEM) combines fluorescence optics with TEM (Agronskaia et al., 2008), the majority are SEMs. One representative ILEM, used here, has a stage with fluorescent optics and an adapter (SECOM-stage, Delmic, Delft, Netherlands, see also Zonnevylle et al., 2013) that couples to a JEOL JSF7200 SEM (Tokyo, Japan).

Successful CLEM with these instruments still requires that samples are prepared and labeled in ways that are compatible with both forms of microscopy. FM is typically done with minimal treatments, so molecules retain their native state and structure, and, as a result, fluorescent proteins retain fluorescence and macromolecules retain antigenicity. In contrast, EM requires multiple treatments that alter macromolecular structures. Firstly, the biological material is primarily composed of chemical elements with low atomic numbers (carbon, oxygen, nitrogen, hydrogen) that poorly scatter electrons and thus provide inherently low EM contrast (Reimer and Kohl, 2008). Consequently, EM imaging is typically performed after biological samples are incubated with compounds containing high-atomic-number elements and that bind to cellular structures like membranes and nucleic acids. Secondly, high-resolution EM requires ultrathin sections of plastic embedded samples. In order to prevent loss of cell-components like proteins, membranes, etc. by extraction during dehydration and infiltration of the plastic, samples are treated to form a crosslinked and stable structure. This requires chemicals like glutaraldehyde and osmium tetroxide, which alter the structure of most macromolecules and strongly quench fluorescence signals in the sample (pre-processing fluorescence; for example, from genetically encoded fluorescent proteins or antibodies labeling). These treatments also impair affinity labeling, like immunolabeling, and thereby reduce post-embedding fluorescent signals (Griffiths, 1993; Watanabe et al., 2011).

Sample fluorescence that remains preserved after embedding is referred to as in-resin fluorescence (IRF). IRF is achievable via high-pressure freezing, freeze-substitution, and the use of acrylic embedding resins such as Unicryl, Lowicryl, or LR White. In particular, Lowicryl resins can be polymerized by UV at sub-zero temperatures, which is beneficial for the preservation of membranes and protein structures (Kukulski et al., 2011; Bharat et al., 2018). With interest in CLEM, their use has been described for cultured cells (Peddie et al., 2014, 2017) and zebrafish embryos (Nixon et al., 2009). It also enabled the correlation of single-molecule localization microscopy and SEM (Johnson et al., 2015). It appears that the presence of water in the substitution medium improves the visualization of membranes (Walther and Ziegler, 2002) and plays a role in the fluorescence of GFP. This latter effect was demonstrated by the re-occurrence of GFP-fluorescence that had faded under high-vacuum upon allowing water to evaporate and enter the high-vacuum chamber of the SEM containing the GFP sample (Brama et al., 2015). This could be compatible with the fact that relaxation of the excited state of GFP is related to a proton transfer chain that includes water (Tsien, 1998). In line with this, reportedly (Peddie et al., 2014), a short freeze-substitution protocol (Mcdonald and Webb, 2011) as opposed to a longer one such as applied to zebrafish embryo’s (Nixon et al., 2009), appears necessary for retaining fluorescence in cells. The much smaller cells would become too dehydrated with the longer FS-times necessary to prepare the larger zebrafish embryo’s optimally. Nonetheless, this subject still contains unknown factors, and successful results have been obtained with longer FS-times and cells (e.g., Kukulski et al., 2011). Here, with this in mind, we tested different sample preparation conditions to optimize IRF, resulting in a CLEM imaging method where IRF is used to define the identity of specific neurons in ultrastructural images of the mouse brain imaged with ILEM.

We also explore how to best use SEM to derive detailed ultrastructure from thin sections. SEM- and ILEM-imaging of thin sections uses backscattered electrons, as this signal discriminates between atoms with high and low atomic numbers. Therefore, biological samples are typically treated with, amongst others, uranyl acetate to achieve this contrast. There are, however, additional parameters that affect image quality. In the past, other groups have obtained results with SEM-imaging of similar preparations using different accelerating voltages and instruments, while information on other parameters was sparse or lacking (Peddie et al., 2014; Markert et al., 2016; Bouwer et al., 2017). More recently, it was shown that biasing the sample can ameliorate SEM-imaging of sections (Vos et al., 2021). In this study, we show how different imaging parameters affect image quality and propose guidelines on how to optimize basic SEM settings in function of section thickness for high-resolution thin-section imaging.

Finally, all of the above considerations and the findings in this paper are equally well applicable to 3D-CLEM by array tomography, whether performing fluorescent and electron imaging on separate equipment or using an ILEM as also used in this study.

## Materials and Methods

### Animals

All animal experiments were approved by the KU Leuven Ethical Committee (protocol P019/2017) and were performed in accordance with the Animal Welfare Committee guidelines of the KU Leuven, Belgium. In total, the brains of two 4 C57BL/6 mice and 2 virus-injected C57BL/6 mice at P21 were used. Mice were euthanized with a mixture of ketamine and xylazine as per institutional guidelines.

The lentiviral vectors harboring a green fluorescent protein (GFP) reporter were kindly provided by Dr. J. de Wit (Schroeder and De Wit, 2018). At P0, mouse pups mice were used for neonatal stereotaxic virus injection. Pups were anesthetized by hypothermia and then stabilized on a glass Petri dish filled with ice to sustain anesthesia during the injection. After disinfection of the injection area with 70% EtOH, 20 nl (around 1,010 transducing units/ml; diluted 1:10 in 1x PBS) of high titer lentivirus with a non-targeting shRNA viral control vector (TTC​TCC​GAA​CGT​GTC​ACG​T) (Wang et al., 2012) was injected with a speed of 4 nl/s directly through the skin and skull using a Nanoject II Auto-Nanoliter Injector (Drummond). Bilateral injections in CA1 hippocampus were made using a depth of 1.1 mm, with two injections per brain hemisphere.

Mice were transcardially perfused with 10 ml of 4% paraformaldehyde (PFA) (#15714, EMS, United States) in 0.1 M phosphate buffer, pH 7.4 (hereafter “PB”) for 10 min and then the brains were postfixed overnight in the same solution at 4°C. Next day, after washing twice in PB, 100 µm-thick coronal vibratome sections of virus injection hippocampus and 80 µm-thick sagittal vibratome sections of the cerebellum were made from. The discs of 1.35 mm of ROI were cut from the vibratome sections of the hippocampus and cerebellum with a punch and submerged in 10% of BSA in 0.1 M PB solution as a cryo-filler.

### Cell Culture

All culture media and sera are from Life Technologies. HeLa cells expressing GFP in the cytoplasm were routinely grown in Dulbecco’s modified Eagle’s medium (DMEM/F12) supplemented with 10% fetal calf serum (FCS) and cultured in 10 cm Petri dishes. All cells were maintained in a humidified chamber with 5% CO2 at 37°C.

HeLa PSEN1/PSEN2 knockout (HeLa dKO) cells were generated with CRISPR/Cas9 genome editing as described (Sannerud et al., 2016). Shortly, HeLa cells were double transfected using JetPrime (Polyplus) with the pX330 plasmid containing guide sequences against the genomic sequence target for both PSEN1 (5′- ATG​AGC​CAC​GCA​GTC​CAT​TC -3′) and PSEN2 (5′-TGT​CAC​TCT​GTG​CAT​GAT​CG-3′). dKO clones were selected by serial dilution. Full knockout was confirmed by genomic sequencing and western blot analysis. For stable GFP expression, lentiviral particles were produced by co-transfection of Hek293 T cells with lentiviral plasmid (pCHMWS-GFP), packaging plasmid (pCMV ΔR8.74) and envelope (pMD2. G) plasmid using JetPRIME (Polyplus). HeLa dKO cells were transduced with these viral particles diluted in normal cell culture medium containing polybrene (8 ng/μl, Sigma). Cells stably expressing GFP were selected with puromycin (3 μg/ml, Sigma) 24 h after transduction.

In order to prevent proteolysis during the variable waiting periods before actual high-pressure freezing, cells were fixed with 4% PFA in 0.1 M PB pH7.4, then washed, scraped and pelleted in the same buffer to a final volume of 1.5 ml. Alternatively, cells were not fixed at first but exposed to 0.05% Trypsin-EDTA (#253000-054, Gibco, United States) to detach from the bottom of the culture plate, washed with 0.1 M PB and pelleted, and then finally fixed. In both cases, after pelleting the cells at 200xg, they were resuspended in 10% BSA (A9647, Sigma-Aldrich) as a cryo-filler, and finally pelleted at 1000xg.

### Immunolabeling

The cerebellar sections were washed three times in 0.1 M PB pH7.4 on ice for 10 min and incubated in 0.3% hydrogen peroxide (H1009, Sigma-Aldrich) in the same buffer for 30 min on ice. After three washes with the same buffer again, sections were incubated in 0.5% sodium borohydride (#71320, Sigma-Aldrich) for 30 min at room temperature, followed by three 10 min washes with 0.1 M PB. The sections were incubated in blocking buffer containing 1% BSA, 0.01% glycine (G7126, Sigma-Aldrich), 0.01% lysin (L5501, Sigma-Aldrich), 1% normal donkey serum (D9663, Sigma-Aldrich), 0.05% Triton X-100 (#22146, EMS), 0.1% cold water fish gelatin (#25560, EMS) in 0.1 M PB for 2 h on ice. Samples were stained with primary antibody, mouse anti-Calbindin-D28 K (#AgCB10 abs, Swant, Switzerland), at 1:5,000 dilution in the same blocking buffer, at 4°C overnight. The following day, slices were washed four times in 0.1 M PB on ice and probed with secondary antibody, donkey anti-mouse conjugated to Alexa Fluor 488 (A-21202, Invitrogen) at 1:200 dilution in blocking buffer for 2 h on ice.

### High-Pressure Freezing (HPF) and Freeze-Substitution (FS)

For high-pressure freezing, cells or discs (1.35 mm diameter) punched out of hippocampal or cerebellar tissue slices (100 µm thick) were loaded in membrane carriers of a HPF (Leica EMPACT2) and vitrified at 2050 bar. The frozen carriers were stored under liquid nitrogen until further processing.

The frozen samples were freeze-substituted in a Leica AFS2 apparatus using our protocol (see below and Table 1) based on the original quick-freeze substitution (QFS) protocol (Mcdonald and Webb, 2011; Peddie et al., 2014), In addition, we constructed a sample holder to hold the samples during the QFS-run, but that fits snugly into the Leica AFS2 apparatus as well, and facilitating the transfer of the carriers from QFS-holder to the flow rings of the AFS (see Supplementary Figure S2).

**TABLE 1:** IRF protocol with freeze-substitution steps after transfer of samples from QFS-box to Leica AFS2 freeze-substitution apparatus. QFS,quick-freeze substitution.

Reagent	Temp	Time	Repeats
Acetone	−50°C	20 min	2x
Ethanol	−50°C	20 min	1x
20, 40, 60, 80% HM20/ethanol	−50°C	45 min	each step 1x
100% HM20	−50°C	8 h	1x
100% HM20	−50°C	2 h	3x
100% HM20 - UV polymerization	−50°C	48 h	1x
100% HM20 - temperature rise with UV polymerization	−50 - 20 °C	16 h	1x
100% HM20 - UV polymerization	20°C	48 h	1x

Briefly, the membrane carriers with samples were transferred to cryotubes (72.694.005, Sarstedt, Germany) with 1.5 ml of freeze-substitution (FS) medium, containing 0.1, 0.2, or 0.5% uranyl acetate (#02624-AB, SPI) and 5% dH_2_O in acetone (#1002990500, Merck), and placed in a QFS-holder. After loading the QFS-holder at −180°C, the liquid nitrogen was removed from the QFS-box, the box was closed and allowed to reach −80°C. After reaching −80°C temperature, the QFS-holder was tipped on its side, the box closed again and agitated on a rotary shaker at 50 cycles/min. The temperature was monitored, and at −50°C, the QFS was stopped and the QFS-holder transferred swiftly to the waiting pre-cooled to −50°C Leica AFS2 apparatus. Our holder fits precisely in the space left in the AFS2 chamber when solution exchange bottles are left out, but flow rings left in place. After transfer of the carriers to the flow rings filled with acetone pre-cooled to −50°C, the QFS-holder is removed from the chamber and solution exchange bottles, filled with acetone, ethanol (#7103, VWR) and Lowicryl HM20 (#02628-AB, SPI), are inserted in the AFS2 chamber. The first bottle of acetone for washing is already pre-cooled to −50°C during transfer of the carriers and the remaining bottles are cooled to −50°C during the first two washing steps with acetone. The samples are kept in their cryotubes with FS medium at −50°C until the time elapsed from the moment the temperature had reached −80°C (start of agitation) had reached 1.5 h. This is comparable with the protocol by Peddie et al. (Peddie et al., 2014), who try to keep the time elapsed from the insertion of the carriers onto the frozen FS-medium (about −150°C) until the start of washing in acetone at −50°C, the same for every FS run at 3 h. In our case, we chose not to take the melting point of acetone (−95°C) as starting point; taking −80°C as a start of this period is more relevant, as at that temperature, the FS- medium can be considered molten and enveloping the samples entirely. Furthermore, the rate of freeze-substitution at −95°C is about 0 in the presence of 5% water, (as we have used to improve membrane preservation and preserve fluorescence), as elegantly demonstrated by Humbel and Müller (1985) by following substitution rate using tritiated H2O release from the sample in the substitution medium. Even at −80°C it takes days to substitute a test strip infiltrated with methylene blue which release in the substitution medium reflects progression of the substitution process as shown earlier by Zalokar (1966). Thus, we take this period starting at −80°C till first washing step as 1.5 h. The remaining steps follow the protocol as described by Peddie (Peddie et al., 2014), except that here ethanol was used for the last step prior to infiltration by Lowicryl HM20 resin and in the infiltration mixtures (see Table 1). Finally, the samples were polymerized at −50°C using UV.

### Microtomy and Imaging

For SEM imaging, serial sections of different thicknesses (50–500 nm) were cut on a Leica Ultracut S ultramicrotome. Sections were collected as ribbons of four to five sections on an ITO-coated coverslip [Pluk et al., (2009) resistivity 15–30 Ohm; #06472-AB, SPI], and glow-discharged in a Leica ACE600 coating unit. For TEM, ultrathin 70 nm sections were cut and deposited on 200 mesh support grids (#01801, Ted Pella, United States).

ITO coverslips with sections were mounted to a holder that could be fitted onto the SECOM stage (DELMIC, Delft, Netherlands), which in turn was mounted to a door that would fit the SEM (JSM 7200F LV, JEOL, Japan), comprising our ILEM. This way, the same area of interest could be observed by fluorescence imaging with a Plan Apo VC 100x oil immersion objective lens with a NA of 1.4 (SECOM), followed by electron imaging (SEM). In several tests (Figures 2–4, Figure 6), sections were imaged by recording BSE signals in a Zeiss VP Sigma SEM and a Gatan OnPoint BSE-detector. When needed for improving EM imaging, after imaging with ILEM-SEM (Figure 7C and Figure 8A–C, left), we post-stained the ITO coverslip with 4% uranyl acetate and Reynold’s lead citrate (Reynolds, 1963) before imaging again the same ITO coverslip (Figure 8C, middle and right).

For observation of ultrastructural details, images were taken with a TEM (JEM1400-LaB6, JEOL, Japan) operated at 80 kV and equipped with an Olympus SIS Quemesa 11 MP camera. Photographs were taken at various magnifications (0.6–20 kx). None of the grids were post-stained.

**FIGURE 1:**
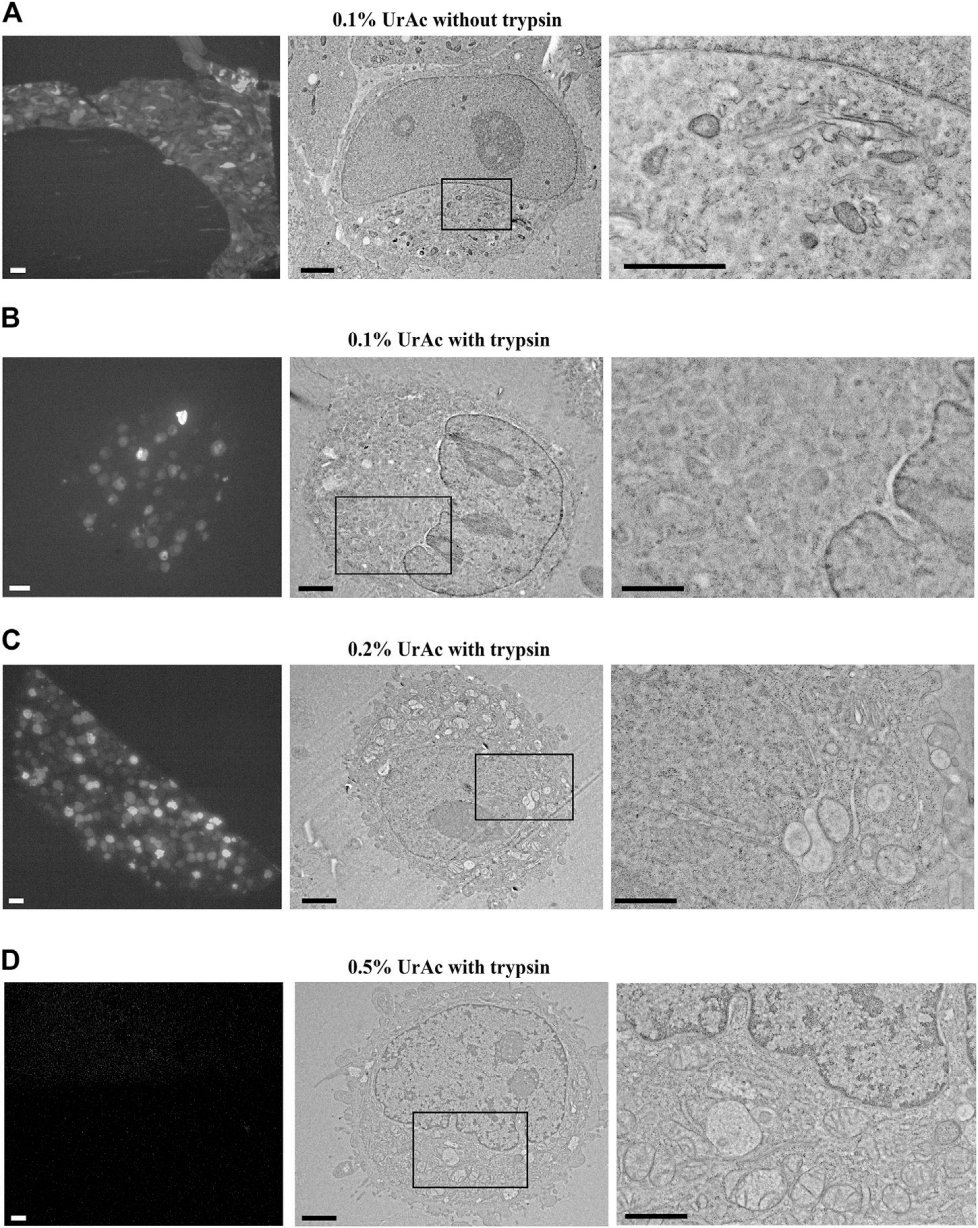
Fluorescence and TEM micrographs of HeLa cells after IRF protocol. **(A)** Cells fixed with 4% PFA and scraped, then processed with 0.1% uranyl acetate; **(B)** Trypsinized cells freeze-substituted with 0.1% uranyl acetate; **(C, D)** Trypsinized cells freeze-substituted and processed with 0.2 and 0.5% uranyl acetate, respectively. Immunofluorescence light microscopy imaging with a Nikon TiE inverted C2 confocal with Plan Apo VC 20x dry lens **(left column)**, TEM at low and high magnification **(resp. middle and right column)**; images in the right column correspond to boxed regions. Note the preservation of fluorescence in conditions **(A–C)** and the best ultrastructure in **(C–D)**. IRF, in-resin fluorescence; PFA, paraformaldehyde; TEM, transmission electron microscopy; UrAc, uranyl acetate. Scale bars: left column = 20 μm; middle = 2 μm; right = 1 µm.

**FIGURE 2:**
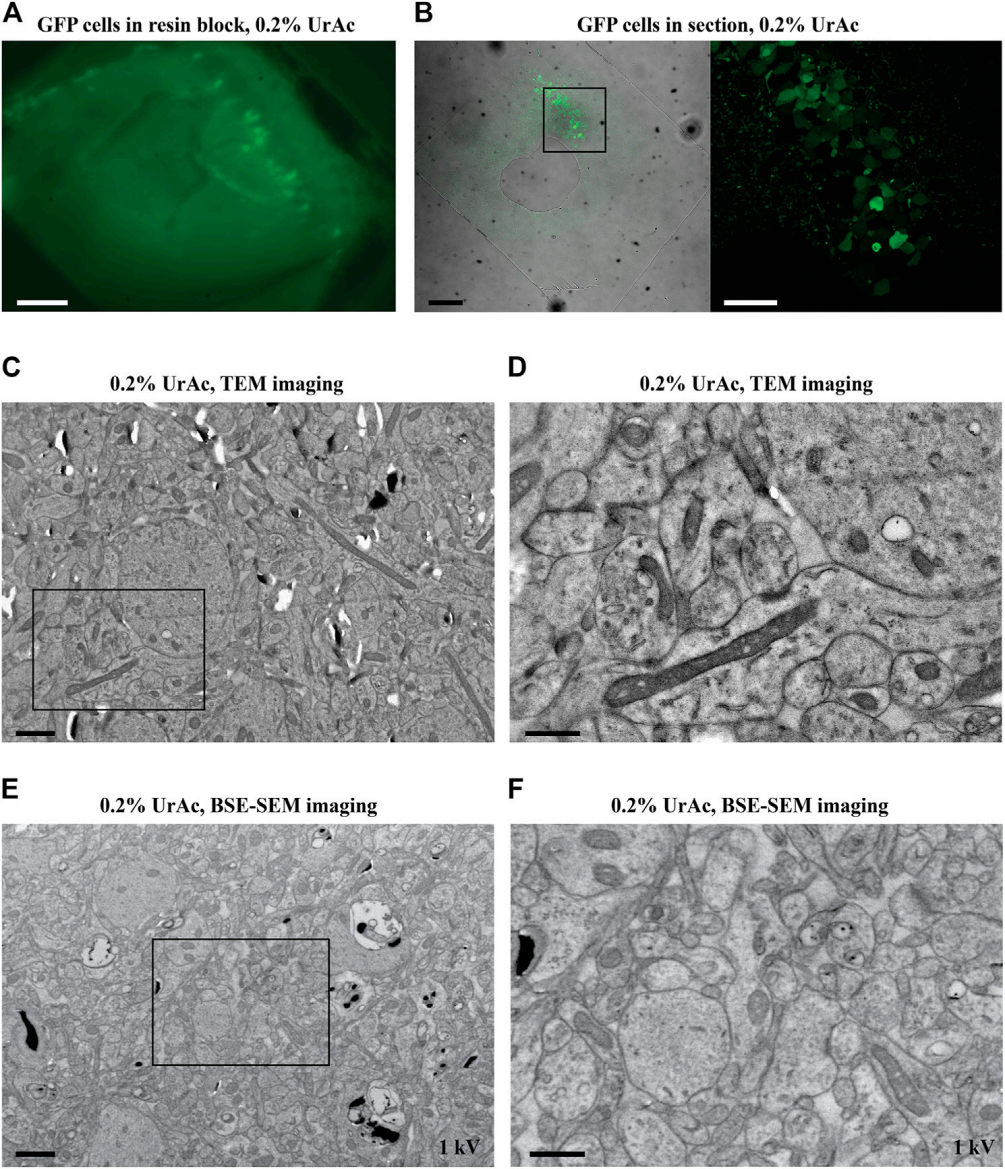
Fluorescence and electron micrographs of mouse hippocampus tissue; IFR protocol freeze-substituted with 0.2% uranyl acetate. The fluorescent signal was preserved in a 100 µm hippocampus slice after IRF protocol as observed in the resin block imaged with widefield Zeiss Axioplan 2 with Plan-NeoFluar 63x lens. **(A)** and after thin sectioning imaged with a Nikon TiE inverted C2 confocal microscope with a Plan Apo VC 20x dry lens **(B, right)** and Plan Apo 10x dry lens **(B, left)**. An enlarged view of the area enclosed in the black square in the left image of **(B)** shows pyramidal cells in CA1 **(B**, **right)**. **(C, D)** TEM imaging of GFP positive CA1 hippocampal cells, showing good ultrastructure. **(D)** A higher magnification image of the area indicated by the black box in **C**. **(E, F)** same sample block as in **(C, D)**, but imaged by SEM using a BSE detector (Gatan OnPoint detector) at 1 kV. The image quality (inverse contrast) is comparable to TEM imaging. WD = 6 mm. Section thickness = 100 nm. Abbreviations: BSE, backscattered electrons detector; GFP, green fluorescent protein; IRF, in-resin fluorescence; SEM, scanning electron microscope; TEM, transmission electron microscopy; UrAc, uranyl acetate; WD, working distance. Scale bars: **A, B** left = 100 μm; **B**, right = 40 μm; **C, E** = 1 μm; **D, F** = 0.5 µm.

For verifying fluorescence in resin blocks we used widefield fluorescent imaging (dry, no coverslip) with a Zeiss Axioplan 2 equipped with a Hamamatsu ORCA-SPARK camera and Plan-NeoFluar 63x lens (Figure 2A). For fluorescent imaging prior to study in the ILEM, we mounted sections on microscopic slides without coverslips and imaged with a Nikon TiE inverted C2 confocal microscope with a Plan Apo VC 20x dry lens (Figures 1A–D, Figure 2B right, Figure 7B) and Plan Apo 10x dry lens (Figure 2B left, Figure 7A).

Probe current measurements in the SEM (Zeiss Sigma) were done at some different imaging parameters. To this end, a stub with a Faraday cup (#651-F, Ted Pella, Redding CA, United States) absorbing all impinging electrons and hence allowing to measure the probe current, was placed in the SEM, and imaging parameters were varied in a range typically used for imaging resin sections with biological material. For all SEM imaging in this study, a standard aperture of 30 µm was used.

### Monte Carlo Simulation

For simulating interaction volumes of electrons and a carbon sample, we used the program Win XRay, (copyright 2002–2015 McGill University), by Demers, Horny, Gauvin, and Lifshin.

## Results and Discussion

### IRF of Cultured Cells

We set out to identify conditions that optimally balance fluorescent signal retention with the preservation and contrasting of cellular structures for EM. As a starting point, we focused on conditions described by Peddie et al. (Peddie et al., 2014), but varying uranyl acetate percentages in the substitution medium for embedding. Importantly, samples were washed with ethanol immediately prior to the first infiltration step with Lowicryl HM20 acrylic resin (see Table 1 for details); a procedure previously reported to improve Lowicryl HM20 resin infiltration into biological samples at the end of a freeze-substitution (Monaghan et al., 1998). As pointed out (see Introduction), the presence of water is important for improved ultrastructure and fluorescence of GFP. In our current set-up, water vapor cannot enter the SEM specimen chamber, hence we verified fluorescence outside the specimen chamber.

HeLa cells expressing green fluorescent protein (GFP) were collected by fixation and scraping or by trypsinization. Scraped cells were lightly fixed (4% paraformaldehyde, PFA) to prevent excessive ultrastructural damage that could be expected to be caused by the scraping procedure. It should be noted that fixation with formaldehyde can still cause artifacts like nuclear granulation (Hayatt, 1981) and “false” septate junctions (Nistal et al., 1978). Nonetheless, for practical reasons, we have opted to use formaldehyde fixed cells to draw a similar baseline for all the samples and relate to our tissue of interest, more specifically brain slices (see below). We tested 0.1, 0.2 or 0.5% uranyl acetate during freeze-substitution. After processing, it appeared that the fixed and scraped cells showed fluorescent signal and ultrastructure in resin sections similar to the trypsinized cells (Figure 1A vs Figure 1B). However, we found that the resin-embedded sections of HeLa cells retained strong fluorescence when treated with 0.1% or 0.2% uranyl acetate (Figure 1A–C, left), whereas the signal was strongly quenched by 0.5% uranyl acetate (Figure 1D, left). In terms of TEM, freeze-substitution with 0.1% uranyl acetate resulted in poor ultrastructural preservation, including discontinuous labeling of membranes and the appearance of empty regions of cytoplasm (Figure 1B, middle and right).

In contrast, many cellular structures like the Golgi apparatus, endoplasmic reticulum, and mitochondrial cristae were more easily identified in samples freeze-substituted in 0.2% uranyl acetate (Figure 1C, middle and right) or 0.5% uranyl acetate (Figure 1D, middle and right). We concluded that 0.2% uranyl acetate provides the best compromise between fluorescence preservation and TEM detection of cellular ultrastructure. However, the tested 0.1% uranyl acetate treatment after fixing cells *in situ* on the tissue culture plates and then scraping (Figure 1A), displayed improved ultrastructural preservation and very well delineated small subcellular structures, even despite the sub-optimal uranyl acetate concentration. Early fixation, therefore, provides additional structural preservation and can compensate for lower uranyl acetate concentrations.

Results from earlier experiments by others (Kukulski et al., 2011) demonstrated that very nice ultrastructural preservation is possible with 0.1% uranyl acetate in the freeze-substitution medium. These results can be explained by some differences with the current study, most notably the long substitution time, but perhaps also the use of TEM (having better resolution and other contrast mechanism than SEM) and post-staining of the yeast cells. At the same time, as will be shown below, we have found that it is important to determine the best match between section thickness and accelerating voltage for BSE-SEM study of sections, and could also obtain reasonable results with 0.1% uranyl acetate (see Figure 4, 1 kV).

### IRF of Brain Samples

We applied the optimized IRF preparation protocol for cells–freeze-substitution in 0.2% uranyl acetate (Figure 1C) to lightly fixed (4% PFA) mouse brain tissue where GFP was expressed in hippocampal pyramidal neurons (Figure 2). We opted for formaldehyde fixation despite the possibility of occurring artifacts as it prevents anoxia-induced ultrastructural changes that start to occur very early on during the sample preparation process. At the same time, formaldehyde fixation will also allow other standard preparation steps, including pre-embedding staining and other procedures on the brain slices before high-pressure freezing. We again saw well-preserved fluorescence: there was a strong fluorescent signal from the resin-embedded tissue block (Figure 2A), as well as the resulting thin sections (Figure 2B). Again, the protocol provided good contrast and preservation of ultrastructure in TEM imaging (Figure 2C) sufficient to resolve synapses, organelles, and other cellular details (Figure 2D). Next, we examined the same samples with our Zeiss Sigma SEM equipped with a backscattered electron detector (BSE; Gatan OnPoint). This SEM allows for relatively rapid navigation and optimization of SEM imaging conditions as compared to the ILEM system. This is because, with the ILEM, stage navigation is controlled by the SECOM stage control software that has no navigation functionalities such as image navigation. The optimization parameters, obtained with the Zeiss SEM and described here, were then used for imaging samples with the ILEM system in this paper. It appeared that this tissue sample also proved to be compatible with good contrast SEM imaging (Figure 2E and Figure 2F), in fact, comparable to that acquired by TEM (Figure 2C and Figure 2D). This observation is contrary to our expectations that − as mentioned in the introduction − for larger samples to combine the preservation of fluorescence and ultrastructure would require longer FS-times than for cells (Peddie et al., 2014). After all, tissue would present a “continuum” of membranes functioning as barriers reducing the diffusion rate of uranyl acetate to the core of the tissue. In the case of cells, the surrounding filler (BSA) would not pose such a barrier. We then re-tested the range of uranyl acetate concentrations in the freeze-substitution medium to determine if further improvement was possible. This was not the case, and results were in-line with the HeLa cell data (data not shown): 0.2% uranyl acetate provides better membrane contrast and definition than 0.1% uranyl acetate, while higher concentrations are incompatible with IRF.

### Tuning SEM Imaging Conditions

There are several ways that SEM image acquisition settings influence image quality, in terms of signal-to-noise ratio, contrast, resolution, as well as sample charging effects. These are poorly investigated for thin section imaging and are important to define, given that they are central to achieving high-quality CLEM with an ILEM. SEM settings also interact in complex ways. For example, higher probe currents elevate the signal-to-noise ratio, but simultaneously also tend to charge the sample, resulting in drift and bad image quality; higher accelerating voltages increase resolution, but typically again at the expense of charging the sample; reduced working distance (WD) improves image resolution, but alters the collection angle for the backscattered or upper electron detectors. On top of these, sample charging is also related to (low) conductivity of the sample, which will cause local heating and hence the expansion of the sample and plastic deformation.

**FIGURE 3:**
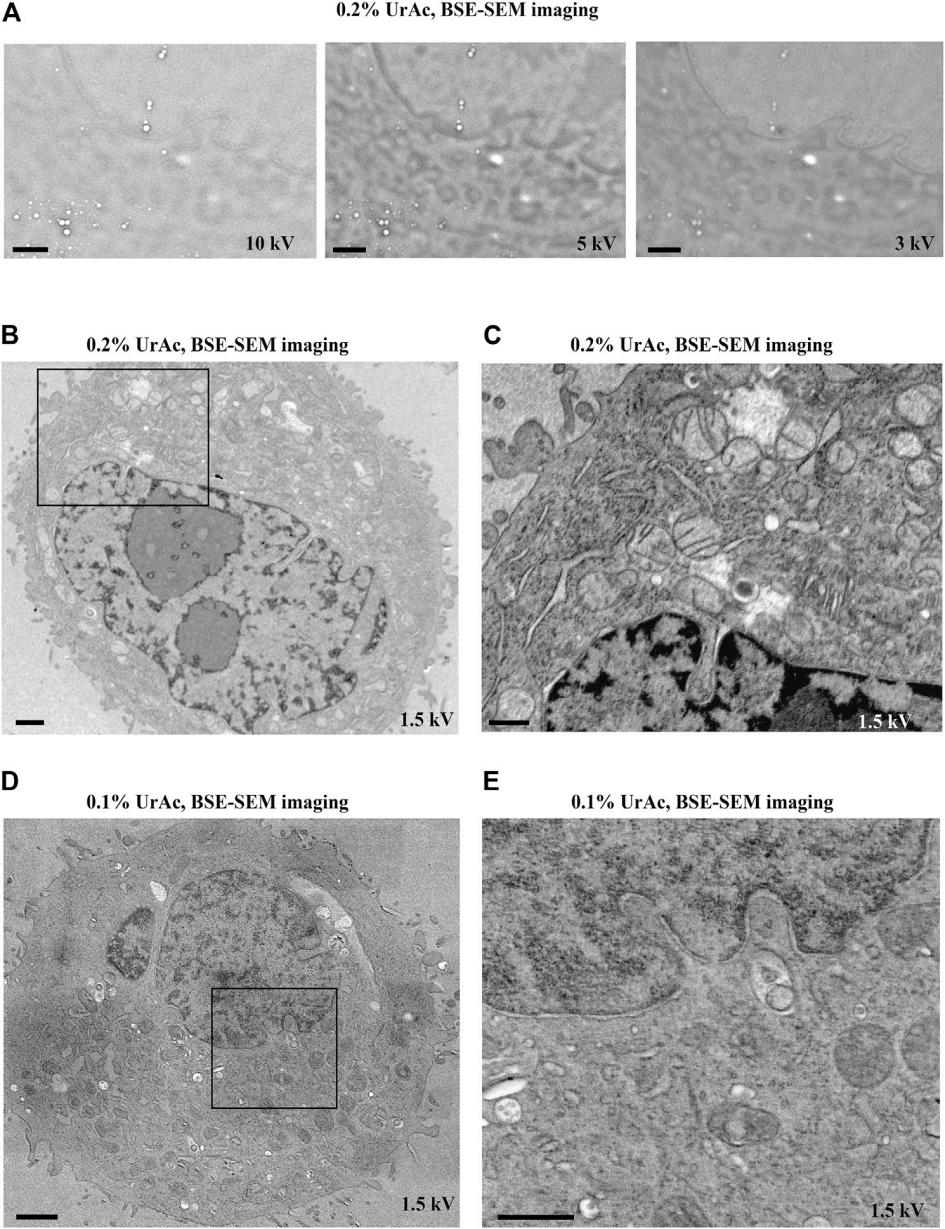
The effect of accelerating voltage and different concentrations of uranyl acetate on image quality (inverse contrast). All images are acquired by SEM and BSE-detector. **(A)** By lowering the accelerating voltage from 10 kV **(A**, **left)** to 5 kV **(A, middle)** to 3 kV **(A**, **right)** imaging of ultrastructural features of trypsinized cells freeze-substituted with 0.2% UrAc gradually improves. **(B, C)** However, the same cells imaged by SEM at 1.5 kV have superior quality, as judged by nuclear details **(B)** and cristae of the mitochondria in an enlarged view of the area **(C)** from the black box in **(B)**. **(D)** Images of representative trypsinized cells freeze-substituted and processed with 0.1% UrAc and imaged with 1.5 kV display a drop in the image quality compared to cells processed with 0.2% UrAc and imaged with same accelerating voltage **(B, C)**. WD **A** left = 6.6 mm, middle = 6.9 mm, right = 7.6 mm; **(B, C)** = 9.7 mm; **(D)** = 9.9 mm. Section thickness = 200 nm. Abbreviations: BSE, backscattered electrons (Gatan OnPoint detector); SEM, scanning electron microscope; UrAc, uranyl acetate; WD, working distance. Scale bars: **(A, B, D)** = 1 μm; **(C, E)** = 0.5 µm.

We first focused on the relationship between accelerating voltage and thin section image quality parameters. This experiment used trypsinized cell samples, freeze-substituted in 0.2% uranyl acetate, that performed well in TEM (Figure 1C). We saw gradual improvements in image contrast as we stepped the voltage from 10 to 3 kV (Figure 3A). Surprisingly, however, the shift from 3 to 1.5 kV (Figure 3A, right vs Figure 3B–C) remarkably improved image quality. On further inspection, we determined that improvements involved more than just altered contrast (Figure 3A); the transition from 10 to 3 kV (Figure 3A) appeared to gradually unveil ultrastructural details, which were then entirely revealed at the 1.5 kV setting (Figure 3B and Figure 3C). We also imaged a sample prepared with 0.1% uranyl acetate at the optimal 1.5 kV accelerating voltage (Figures 3D,E). In this case, we saw low-contrast, poorly defined membranes, alongside some charging (Figure 3C vs Figure 3E), suggesting that the concentration of high atomic number elements has a role in the background signal.

We reasoned that the as yet unknown masking of ultrastructural details (Figure 3A) might relate to charging of the samples. The charging of a sample occurs when not all of the electrons received by the sample can be conducted to ground or scattered by the sample, resulting in a build-up of a negative charge. This in turn repels incoming electrons and saturates the electron detector; images taken of charged samples typically show very bright areas and white streaks. Thus, sample charging relates to the electron dose received per unit area on the one hand and to the conductivity of the sample and quantity of scattered electrons on the other hand. First, we will address electron dose, which is affected by a number of imaging parameters. These include probe current, which, in turn, is affected by the accelerating voltage and can be varied independently to a smaller or larger extent on some instruments, e.g., our Zeiss Sigma and JEOL JSM7200 SEM, respectively. Along the same lines, the dwell time per pixel, and pixel size (in turn, related to magnification), may also affect sample charging. Hence, for a certain probe current, longer dwell-times will result in a higher electron dose, and for a certain probe current—dwell time combination, smaller pixel size will increase the electron dose as well.

We first investigated the relation between probe current and accelerating voltage (see Materials and methods), and found very similar electron doses across the range of tested accelerating voltages: 900 electrons per nm^2^ with 1–5 kV, and 1,200 electrons per nm^2^ at 10 kV (Table 2, example 1–5). It should be noted that at higher accelerating voltages (>10 kV), the electron dose increases notably (see Supplementary Figure S1). Data in Table 2 were obtained at WD between 5 and 6 mm, at which we did all our SEM-imaging, but in this range, the probe current hardly changes, as is also apparent from Supplementary Figure S1.

**TABLE 2:** The effect of some imaging parameters on electron dose. Mag, magnification; Pxl, pixel size; Acc V, acceleration voltage; WD, working distance.

Image Name - Mag	WD [mm]	Pxl [nm]	Acc V [kV]	Dwell time [µs]	Probe current [pA]	Electron dose [electrons per nm^2^]	Effect
example 1–25 K x	5	8	1	50	180	870	No effect with increasing Acc V
example 2–25 K x	5	8	1,5	50	180	870	
example 3–25 K x	6	8	3	50	175	840	
example 4–25 K x	6	8	5	50	186	900	
example 5–25 K x	5	8	10	50	249	1,200	Little effect with higher Acc V
example 6–25 K x	6	8	5	256	186	4,590	Big effect with higher/lower dwell time
example 7–25 K x	6	8	5	8	186	140	

**FIGURE 4:**
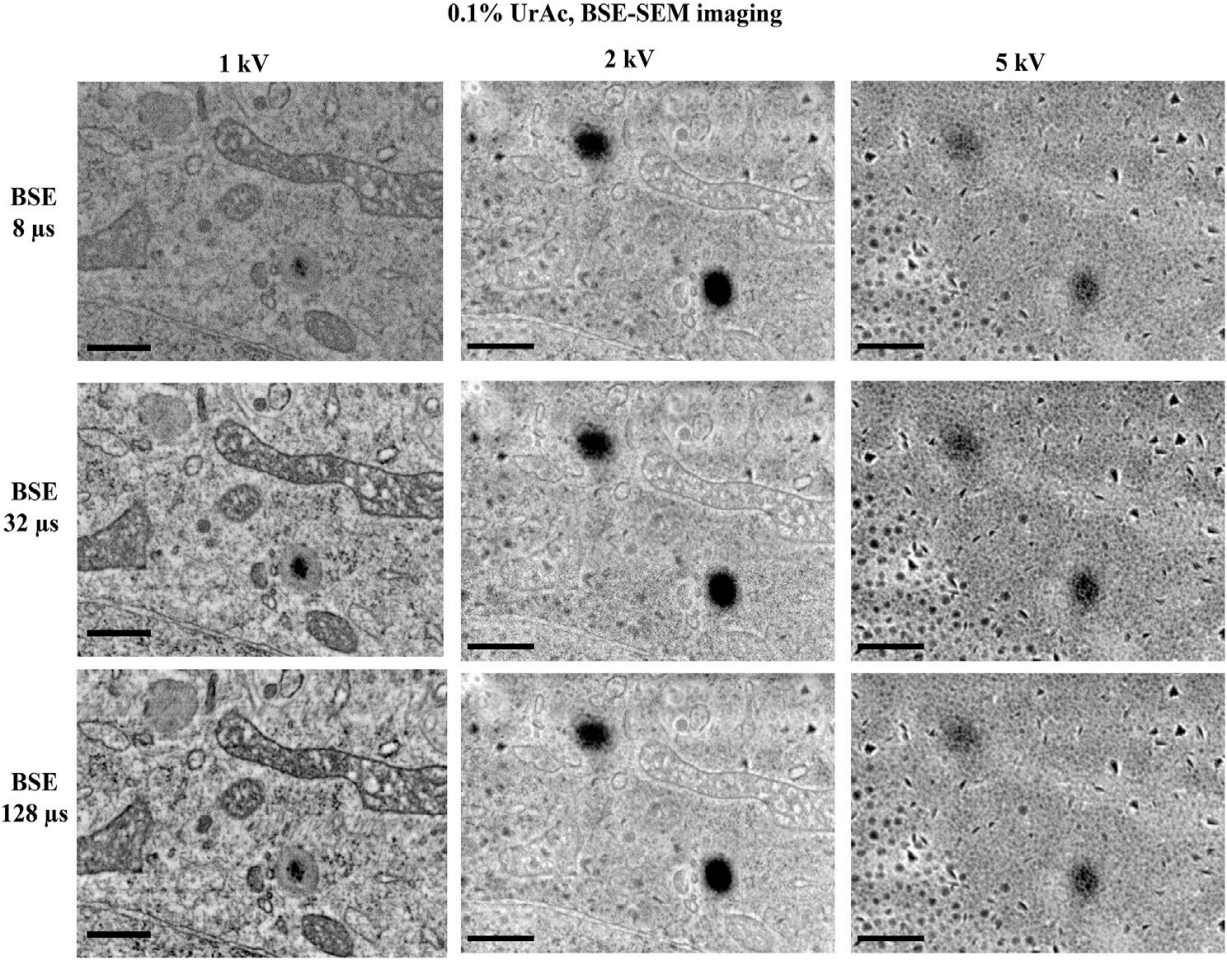
The effect of varying dwell time on image quality. All images are taken from trypsinized cells freeze-substituted and processed with 0.1% uranyl acetate, and imaged by SEM and BSE-detector at an accelerating voltage of 1, 2 and 5 kV. Clearly the image quality was optimal at 1 kV with well resolved mitochondrial details and nuclear envelope. However, at 2 kV the image started to display blob-like structures and at 5 kV no cellular ultrastructure could be discerned; only the blobs were very clear. Reducing the dwell time from 128 µs (third row) per pixel to a mere 8 µs (top row) per pixel has no major effect on the image quality, at none of the tested accelerating voltages. WD 1 kV = 6.1 mm; 2 kV = 4.7 mm; 5 kV = 4.1 mm. Section thickness = 150 nm. Abbreviations: BSE, backscattered electrons (Gatan OnPoint detector); WD, working distance; UrAc, uranyl acetate. Scale bars: 0.5 µm.

The measurements also show how dwell times–expectedly–affect electron dose (Table 2, example 6 and 7: a 32-fold decrease in dwell time proportionally affects electron dose). We, therefore, examined whether dwell times caused sample charging and thus accounted for masking of ultrastructural details. However, the high level of masking of ultrastructural details in images acquired at a 5 kV accelerating voltage was similar when these were acquired at a 128 µsec, or an 8 µsec per pixel dwell time (Figure 4). Moreover, images acquired at 1 kV accelerating voltage were all of high quality, regardless of dwell time. Only at 8 µsec per pixel the contrast dropped (because of too low signal). It, therefore, seems that masking of ultrastructural details that appears in SEM thin section imaging is unrelated to sample charging by an increased electron dose.

As far as the other factors potentially responsible for sample charging are concerned, sample conductivity does not change in function of imaging parameters, as the same area was imaged in these experiments. Moreover, higher accelerating voltages may be expected to decrease the probability of sample charging, as will become clear in the next section. This is in contrast with the finding that image quality gets worse with higher accelerating voltage. Hence, increased accelerating voltage within the range used in our experiments, does not affect image quality through charging, neither through increased electron dose nor by the decreased quantity of escaping electrons.

### Monte Carlo Simulations for SEM Imaging Conditions

We next hypothesized that image quality—as defined here as “containing well discernable cell-structural information” is determined by an interplay of section thickness and accelerating voltage. This is based on considering that the regions of interest are imaged by backscattered electrons, reflected by the high atomic number metals added to the sample. They originate from deeper regions in the section. Another class of electrons, secondary electrons (SE), originate from a more superficial layer of the section and primarily informs us on the topography of the section. Increasing the acceleration voltage also increases the depth of penetration of the electrons into the section. Hence, it might well be that for a certain section thickness, there will be a maximum acceleration voltage above which the contribution of backscattered electrons will be marginal, as they would originate from regions below the section.

**FIGURE 5:**
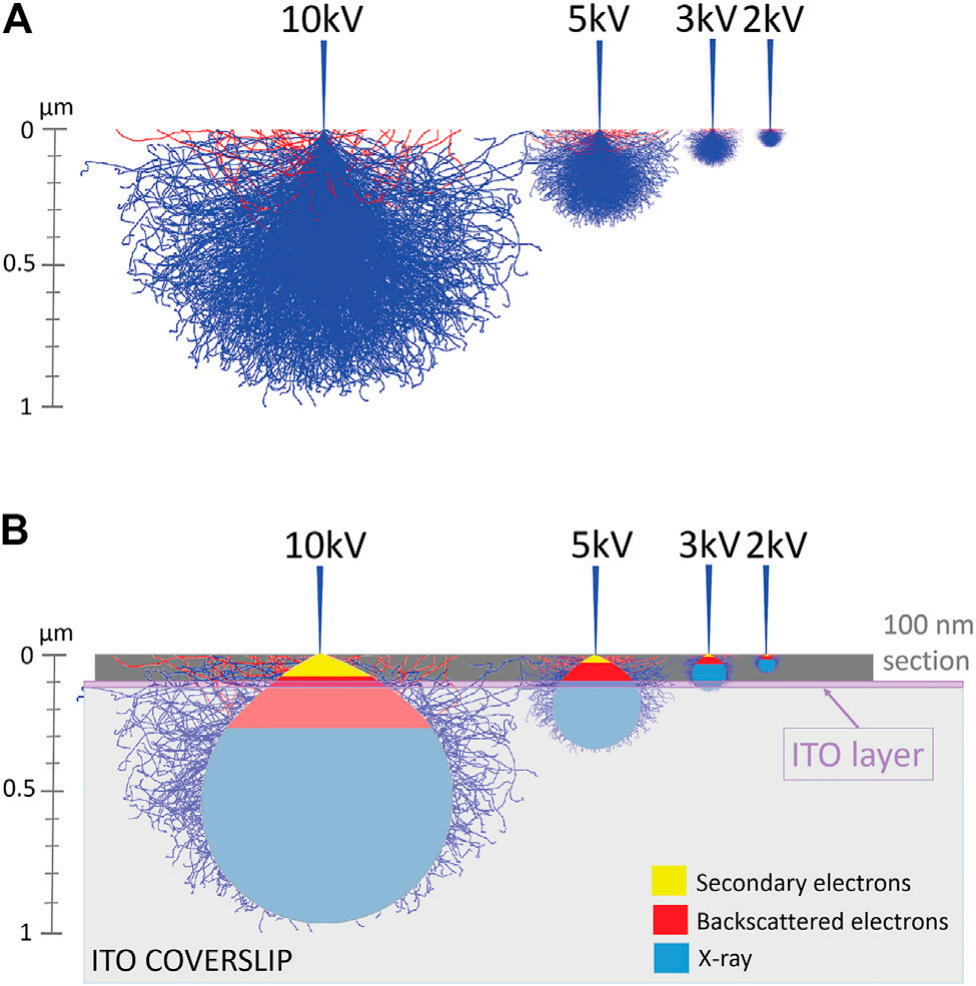
Monte Carlo simulations of the interaction volume of electrons and carbon, demonstrating the relationship between penetration of electrons in a sample and accelerating voltage of the SEM. **(A)** Simulated interaction volumes in carbon sample in function of accelerating voltage of the SEM. **(B)** A schematic indication of the regions of interaction volume that produce secondary electrons (yellow region) and backscattered electrons (red region) is added. Abbreviations: ITO, indium-tin-oxide; SEM scanning electron microscope.

Therefore, we performed mathematical Monte Carlo simulations to predict an “interaction volume” where entering electrons interact with atoms in a sample. Thus, we simulated interaction volumes with samples composed of carbon and respectively 0, 1, 10, and 100% uranium. Interestingly, we find that even up to 10% uranyl acetate, the interaction volume does not differ from simulations with lower percentages of uranyl acetate. On the other hand, samples containing 100% uranyl acetate displayed a markedly smaller interaction volume (see Supplementary File MC simulations). He et al. (2018) estimated maximally 3% heavy atoms in the most heavily stained areas of a very strongly contrasted piece of liver tissue. In comparison, our preparation (max. 0.2% uranyl acetate) can hardly be called “heavily stained” and therefore, the interaction volumes for 0–10% can be regarded as representing our samples. Figure 5 demonstrates how this interaction volume is affected by accelerating voltage. At 10 kV, electrons interact with sample atoms to a depth of about 1 μm, and at 2 kV only of 100 nm. Thus, for thin sections, higher accelerating voltages beam electrons through the sample to the indium-tin-oxide (ITO)-coating of the coverslips used in SEM and/or ILEM system. As seen in Figure 5B, imaging a 100 nm section at 10 kV, the entering electrons primarily generate secondary electrons (yellow) in the sample section, and there are a few backscattered electrons-producing interactions (red). At 5 kV, backscattered electrons are also generated in the section, but some of these penetrate through the section and will be dissipated by the ITO-layer.

In contrast, at 3 and 2 kV, all backscattered electrons arise from interactions in the thin section, thus producing the highest backscattered-/secondary electron (BSE/SE) ratio and maximal compositional information. At too low accelerating voltages, the interaction volume will not reach the ITO layer and may cause charging (Figure 5B, see 2 kV). The simulation can explain why our thin section images had less cellular information if acquired at a 10 kV compared to 3 kV accelerating voltage (Figure 3A). Our hypothesis is supported by the observation mentioned above, that masking of ultrastructural details observed in the BSE-image disappeared by lowering the accelerating voltage from 5 to 1.5 kV (100 nm section) (Figure 3).

**FIGURE 6:**
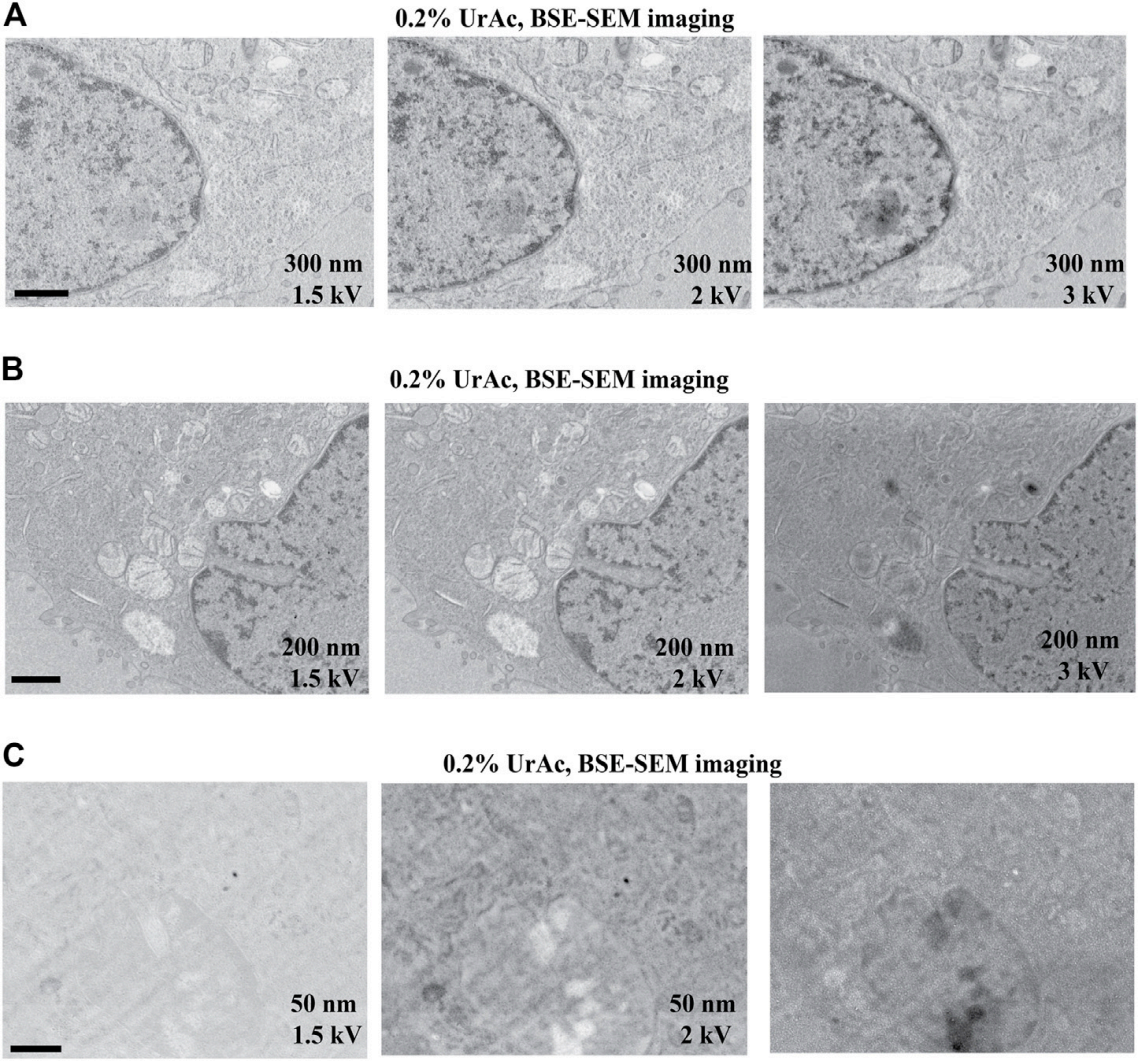
Image quality as function of section thickness (vertical) and accelerating voltage (horizontal). All images represent trypsinized cells freeze-substituted with 0.2% UrAc and sectioned at 300 nm **(A)**, 200 nm **(B)** and 50 nm **(C)**. All images are acquired by SEM and BSE-detector. The thicker the section, the higher the tolerated accelerating voltage is before quality breaks down. WD A 1.5 kV = 5 mm; A 2 kV = 4.6 mm; A 3 kV = 4.3 mm; B 1.5 kV = 5.1 mm; B 2 kV = 4.7 mm; B 3 kV = 4.4 mm; C 1.5 kV = 5.2 mm; C 2 kV = 4.8 mm; C 3 kV = 4.5 mm. Abbreviations: BSE, backscattered electrons (Gatan OnPoint detector); SEM, scanning electron microscope; UrAc, uranyl acetate; WD, working distance. Scale bars: 1 µm.

We further experimentally tested the prediction from the model that thinner sections would require lower accelerating voltages (Figure 6 and Supplementary Figure S3). For 300 nm sections, we collected good quality images at 1.5 kV (Figure 6A, left), 2 kV, and 3 kV (Figure 6A, middle and right). For 200 nm sections, 1.5 kV (Figure 6B, left) and 2 kV (Figure 6B, middle) generated high contrast images, while quality deteriorated at 3 kV (Figure 6B, right). In contrast, all tested accelerating voltages imaged a 50 nm section poorly (Figure 6C and Supplementary Figure S3). Thus, it appears that the thinner the section, the lower the acceleration voltage at which this “image quality breakdown point” occurs. In fact, 5 kV was also effective, and interfering masking of ultrastructural details only appeared at a 10 kV accelerating voltage (Supplementary Figure S3). For example, in 300 nm sections, “image quality breakdown point” occurs at 5 kV, while in 50 nm sections, it occurs at 1.5 kV (see Table 3 and red demarcating line in Supplementary Figure S3). As repeated imaging at different accelerating voltages of the same area will deteriorate the image, the sequence of imaging was reversed without any relevant effects (data not shown). Moreover, in Supplementary Figure S3 the imaging sequence in each section was 3, 2, 1.5, 1, 5, 10 kV. If masking of ultrastructural details were due to repeated imaging the same region, deterioration would be expected in this order as well. However, in particular in case of the 100 nm section, we see an opposite effect; from 3 to 1.5 kV the image quality improves. Our data imply a relationship between acceleration voltage and section thickness. The exact values of these parameters will depend on the experimental setup used, for instance, different BSE-detectors, different SEM, etc. Also, the type of substrate—silicon wafer, Kapton tape - may affect the outcome; in this respect, it may be relevant that BSE detectors may display some sensitivity to cathodoluminescence, invoked by the interaction of electron beam and ITO. Nonetheless, these observations confirm our prediction and support our hypothesis that image quality (observed in Figure 3A) was compromised by an interaction between section thickness and acceleration voltage.

**TABLE 3:** Section thickness vs. Image quality breakdown point. The latter is the accelerating voltage in kilovolt (kV) at which the quality of the image starts to degrade for a certain section thickness in nanometer (nm). Higher accelerating voltages yield worse image quality.

Thickness [nm]	Image quality breakdown point [kV]
**50**	**1.5**
**100**	**2**
**200**	**3**
**300**	**5**
**500**	**10**

### Imaging with ILEM-SEM for simultaneous fluorescence and electron signal detection.

**FIGURE 7:**
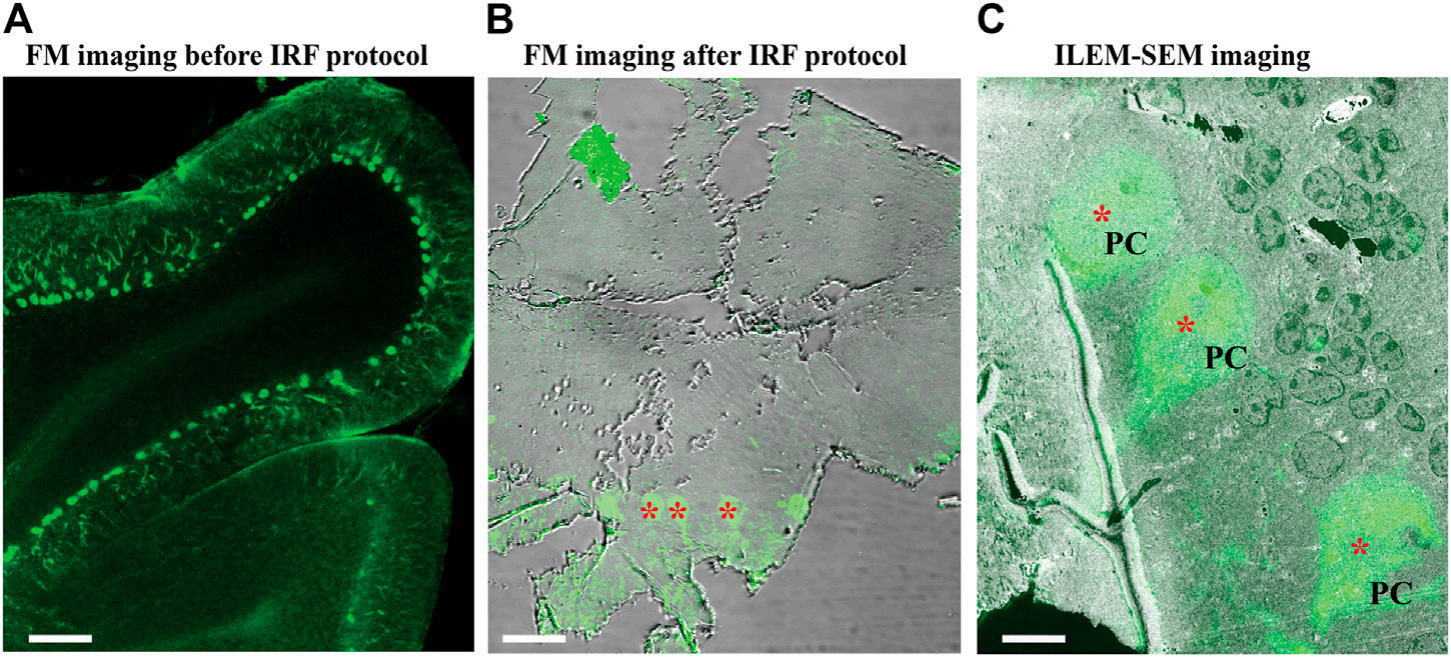
Fluorescence images of immuno-labeled mouse cerebellum imaged with conventional FM and ILEM system (JEOL BSE detector). **(A)** An 80 µm slice of cerebellum labeled with calbindin D28 K primary and Alexa 488-conjugated secondary antibody before embedding with our IRF protocol. Imaged with conventional FM Nikon TiE inverted C2 confocal microscope Plan Apo 10x dry lens, labeled Purkinje cells can be easily recognized (asterisks). **(B)** After embedding with our IRF protocol, 150-nm cerebellar section imaged by conventional FM Nikon TiE inverted C2 confocal microscope Plan Apo 20x dry lens shows that the fluorescent labeling of Purkinje cells (asterisks) was preserved after embedding. **(C)** The same 150 nm section with a layer of Purkinje cells **(**asterisk, in **B)** imaged with fluorescent optics of ILEM-SEM (Plan Apo VC 100x lens) system shows a perfect correlation. Abbreviations: FM, fluorescence microscope; ILEM-SEM, integrated light and electron microscope; IRF, in-resin fluorescence; PC, Purkinje cell. Scale bars: **A** = 100 μm, **B** = 10 μm, **C** = 5 µm.

Finally, we further optimized these protocols for imaging with a simultaneous fluorescence and electron signal system (ILEM-SEM) and antibody labeling as a fluorescence source. We collected 80 µm thick sections of fixed mouse brain cerebellum using a vibratome. The cerebellum is a complex brain area that contains many different cell types. We labeled the sections with an antibody against the protein calbindin D28 K and an Alexa Fluor 488 conjugated secondary antibody (Boey et al., 2019). Calbindin is specifically expressed by Purkinje cells in this brain area, and thus this labeling defines cells with this cellular identity. We also confirmed with conventional FM imaging that Alexa Fluor 488 signal was localized to the cytoplasm and dendrites of Purkinje cells present in vibratome sections (Figure 7A). We then applied our IRF protocol−the freeze-substitution method with 0.2% uranyl acetate and Lowicryl HM20 embedding−to the tissue, followed by mounting ribbons of consecutive 150 nm sections onto ITO coverslips. The thin sections were re-examined in FM to confirm that fluorescent signals had been preserved (Figure 7B). We then selected a region that contained three fluorescent Purkinje cells (Figure 7B, asterisk) and imaged it at low magnification with our ILEM-SEM (Figure 7C, asterisk).

**FIGURE 8:**
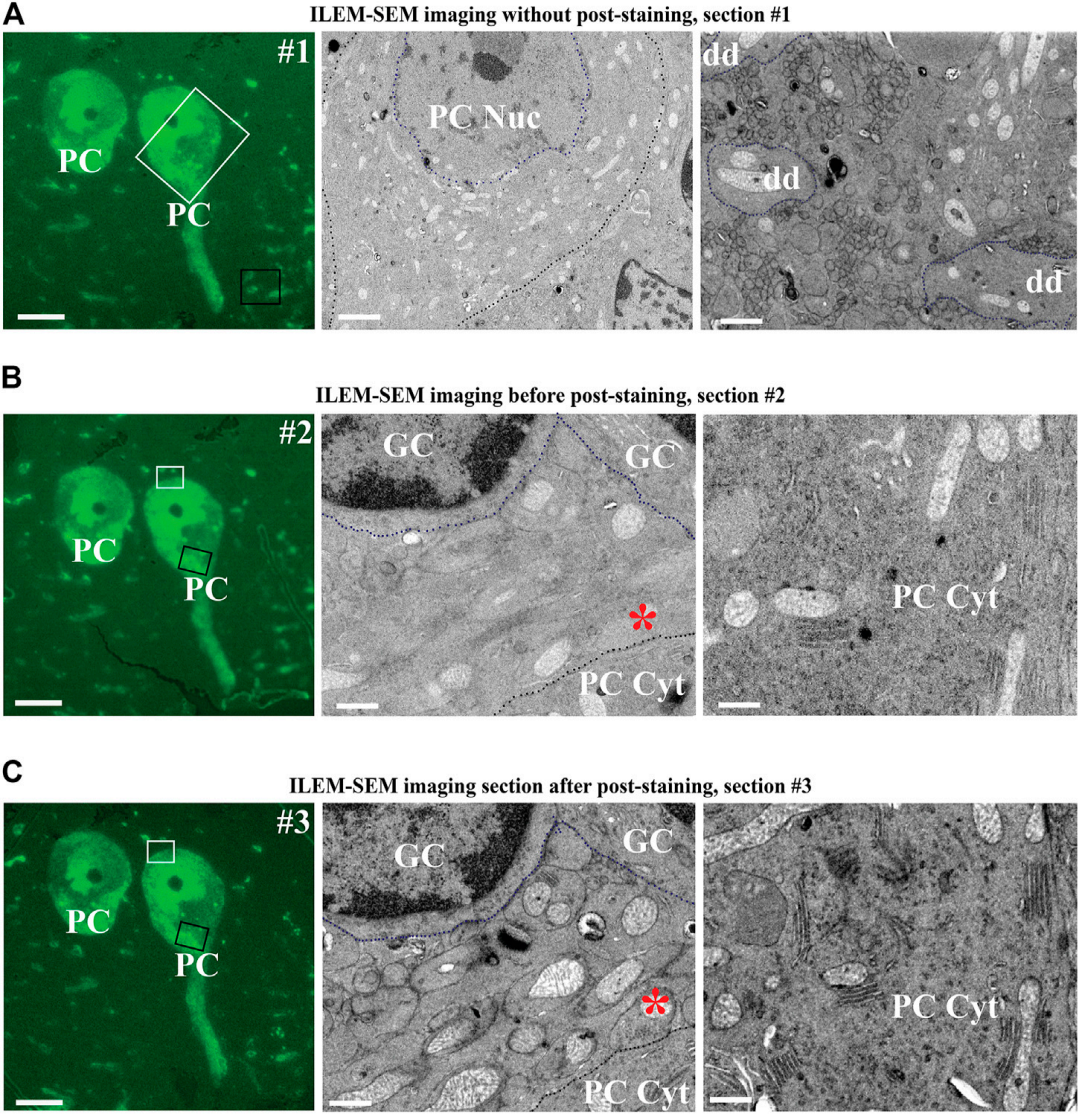
Three consecutive sections of mouse cerebellum immunolabeled for calbindin D28 K and imaged with ILEM-SEM system (JEOL BSE detector). **(A–B,** **left)** Same two large fluorescent Purkinje cells as in the center of Figure 7C, in two immediately adjacent consecutive 150 nm sections without any additional post-staining, imaged at higher magnifications with ILEM-SEM in FM mode with Plan Apo VC 100x lens. **(A–B, middle and** **right)** ILEM EM mode images indicated by white and black boxes in **A–B,** left. Purkinje cell boundaries **(A–B**, **middle)**, granular cells **(B**,** middle)** and three cross-sectioned dendrites **(A**, **right)** are demarcated by a dotted line for ease of recognition. **(C**, **middle and** **right)** Higher magnifications ILEM-SEM images from the regions indicated by white and black boxes in **(C,** **left)**. The images in **C** **(middle** **and** **right)** represent next consecutive section at the same place as in 8 **B** **(middle** **and** **right)**, but after post-staining with uranyl acetate and lead citrate. Post-staining enhanced contrast, so that the mitochondria, membranes, the secretory pathway organelles in PC cytoplasm, and climbing fiber synapse **(**asterisk in **B** **middle** as compared **C** **middle)** are now well distinguished. Abbreviations: Cyt, cytoplasm; dd, dendrite; ILEM-SEM, integrated light and electron microscope; IRF, in-resin fluorescence; GC, granular cell; Nuc, nucleus; PC, Purkinje cell. Scale bars: **(A–C),** left = 10 μm; **(A–C)**, middle = 0.6 µm; **(A–C)**, right = 0.8 µm.

Subsequently, we reselected two cells to evaluate higher magnification imaging using ILEM in FM-mode and then in EM mode (Figure 8). We could easily identify the same two fluorescent cells in the next three consecutive sections (Figure 8A–C, left). Next, by using ILEM in EM-mode, we observed details of the boxed areas, for example, in Figure 8A, representing an area containing the nucleus of the Purkinje cell (white box in Figure 8A left FM mode and middle EM mode) and three cross-sectioned dendrites (black box in Figure 8A left FM mode and right EM mode). Those images demonstrate that fluorescently marked cellular structures can be identified and studied at the ultrastructural level using an ILEM-SEM system.

Notably, the ultrastructural details were less well-defined than typical for TEM, especially when examining smaller subcellular organelles. In addition, a difference with BSE-SEM images of brain tissue obtained with the Zeiss Sigma with OnPoint detector (Figures 2C–E) could be observed. This difference could be due to different factors, including the type of BSE-detector, and the pre-embedding immuno-labelling. Nonetheless, we tested whether further improvement was possible by post-staining with 4% uranyl acetate and Reynold’s lead citrate. We imaged the section before post-staining by ILEM-SEM (Figure 8B middle and right), where it was possible to delineate the type of the cells and larger organelles like the nucleus and mitochondria, but without fine detail, and smaller organelles were indistinct (Figure 8B right). We then re-imaged the next consecutive section after performing a post-staining (Figure 8C). There was a striking improvement of contrast and definition: unlabeled non-fluorescent granular cells could be recognized more easily (Figure 8B, middle vs. Figure 8C, middle), the cristae of mitochondria (Figure 8B, middle and right as compared to Figure 8C, middle and right) and climbing fiber (synapse asterisk in Figure 8B, middle as compared to Figure 8C, middle), contacting the fluorescently labeled Purkinje cell, could be clearly discerned. It was also possible to delineate secretory pathway organelles in the Purkinje cell cytoplasm (Figure 8B, right vs Figure 8C, right). In addition, we could observe by CLEM on some high magnification areas in these cells, that brighter spots in the Soma correspond to lysosome-like organelles (Supplementary Figure S4), indicating a higher concentration of calbindin in these organelles as compared to the surrounding cytoplasm. This observation is analogous to what others have found in intestinal endothelial cells (Nemere et al., 1991). With respect to imaging goals with our ILEM-SEM, the intention is to obtain correlated image sets. In a first round, this will be achieved by combining preserved fluorescence images with corresponding lower contrast unstained EM-images. Then, after the image contrast has been improved by post-staining, the navigation parameters determined and stored by our navigation software (Gabarre et al., 2021) during the first round will be re-used to automatically re-acquire the same but contrast-enhanced set of correlated EM-images. Thus, this two-step ILEM-SEM procedure allows defining cellular identity using antibody labeling and FM, followed by high resolution, fine ultrastructural imaging of subcellular contents and relationships with surrounding cell types.

## Conclusion

In this study, we describe a procedure to prepare GFP expressing cells or tissue and fluorescent antibody-labeled tissue for CLEM, emphasizing the preservation of fluorescent signals. This procedure involves high-pressure freezing and introducing of a freeze-substitution IRF protocol with embedding in Lowicryl HM20 resin. We found that a concentration of 0.2% uranyl acetate in the freeze-substitution medium is optimal for preserving in-resin fluorescence as well as ultrastructure in cells and can be applied successfully for the same purposes to brain tissue with sufficient contrast. It is of great importance to know how to select optimal imaging conditions for visualizing different types of sample with BSE-imaging in an SEM. Bouwer et al. (Bouwer et al., 2017) proposed applying a negative bias voltage to the sample to decrease the interaction volume for a given acceleration voltage and improve sectioning capabilities in block-face SEM, where the block face is imaged. More recently, Vos et al. (2021) and Lane et al. (2021) extended this approach to BSE-imaging of sections in a SEM. Although very useful, this condition is not available to many SEM-users. Therefore, in this paper, we focus on BSE-imaging sections without applying such a bias voltage, which is relevant for array tomography (Micheva and Smith, 2007; Oberti et al., 2010; Collman et al., 2015) in the context of optimizing image quality. Our experiments with different parameters defining imaging conditions in the SEM showed that an important factor determining image quality is the relation between section thickness and accelerating voltage. It appears that for a given section thickness, the image quality diminishes when increasing the accelerating voltage above a certain value. The thicker the section, the higher this value. We have shown that charging of the section is not responsible for this diminished image quality. However, these observations can be explained by considering the interaction volume of electrons and matter, where the ratio BSE/SE-signal will diminish at “image quality breakdown point”, increasing the proportion of surface information (including defects such as knife marks, surface roughness etc.), masking pertinent biological information. Our observations are very important when considering three-dimensional CLEM experiments, such as array tomography, aimed at constructing a correlated fluorescence and electron three-dimensional reconstruction of a sample. For higher resolution reconstructions, the limiting factor in array tomography is the Z-resolution or the section thickness. Improvement of this resolution requires thinner sections. In our studies, it appeared that 50 nm sections require accelerating voltages not higher than 1 kV.

In conclusion, we have tuned a protocol for the preservation of fluorescent signals present in cells and tissue samples, as well as their ultrastructure, and determined optimal conditions for BSE-imaging with ILEM system. This advancement is important for transitioning from two-to three-dimensional CLEM imaging in an automated fashion with a new generation of ILEM systems.

## Data Availability

The raw data supporting the conclusion of this article will be made available by the authors, without undue reservation.
