# Supplementary figures and images for: Preservation of Fluorescence Signal and Imaging Optimization for Integrated Light and Electron Microscopy
Source: Front Cell Dev Biol. 2021 Dec 15;9:737621. doi: 10.3389/fcell.2021.737621 (PMC8715528; doi:10.3389/fcell.2021.737621)

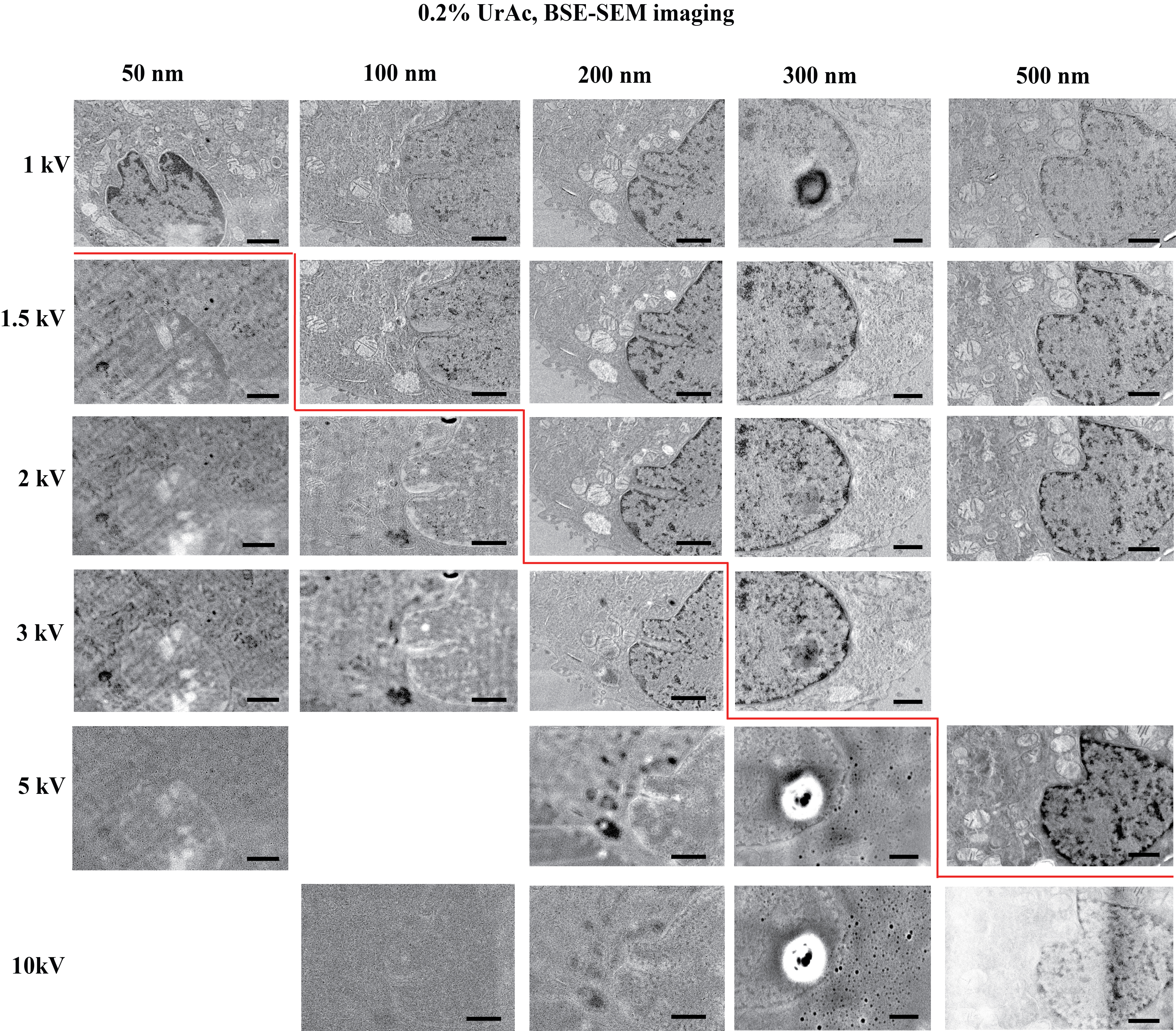

Supplement: Supplementary file 1 [file Image3.JPEG]

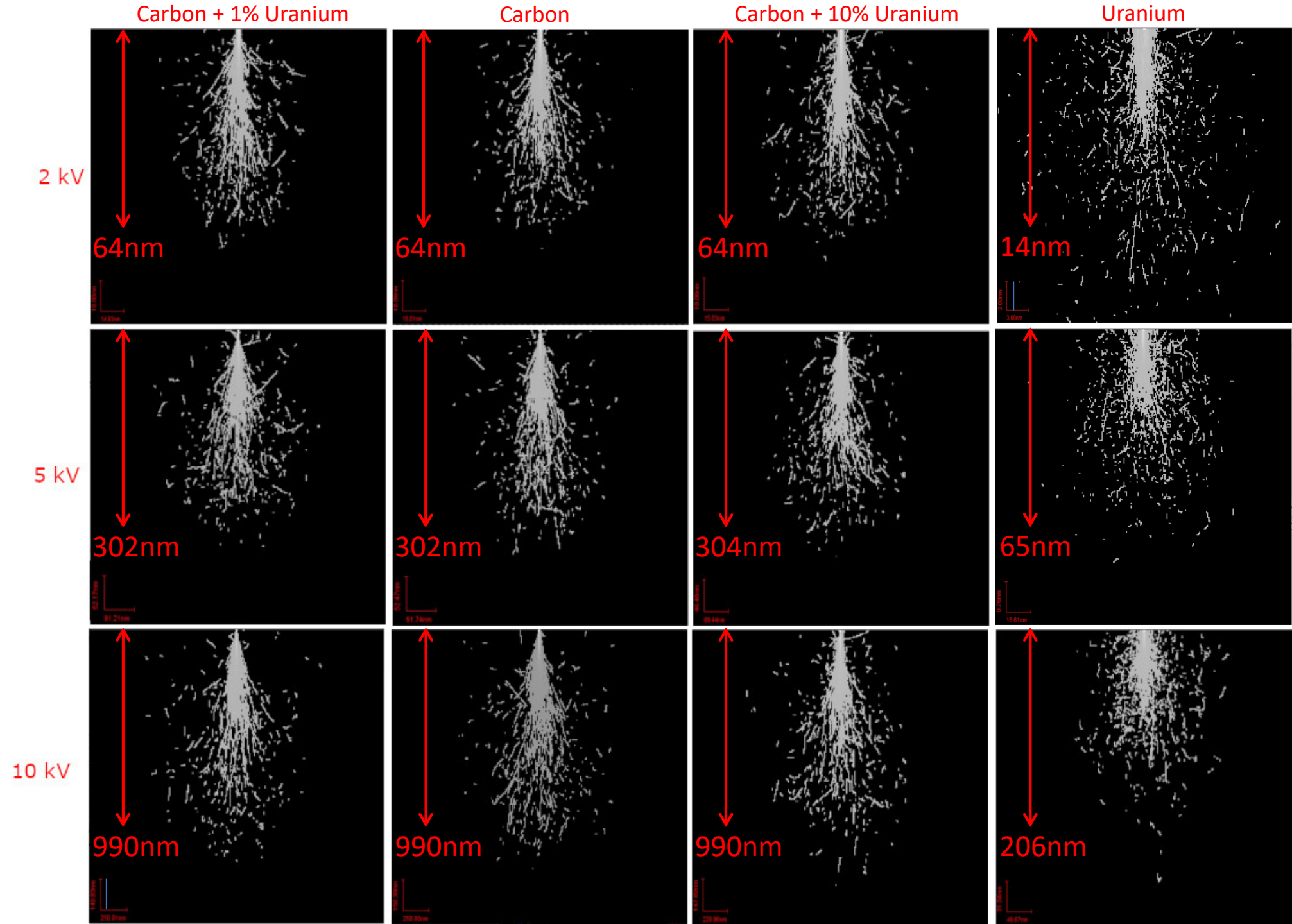

Supplement: Supplementary file 2 [file Image5.pdf]

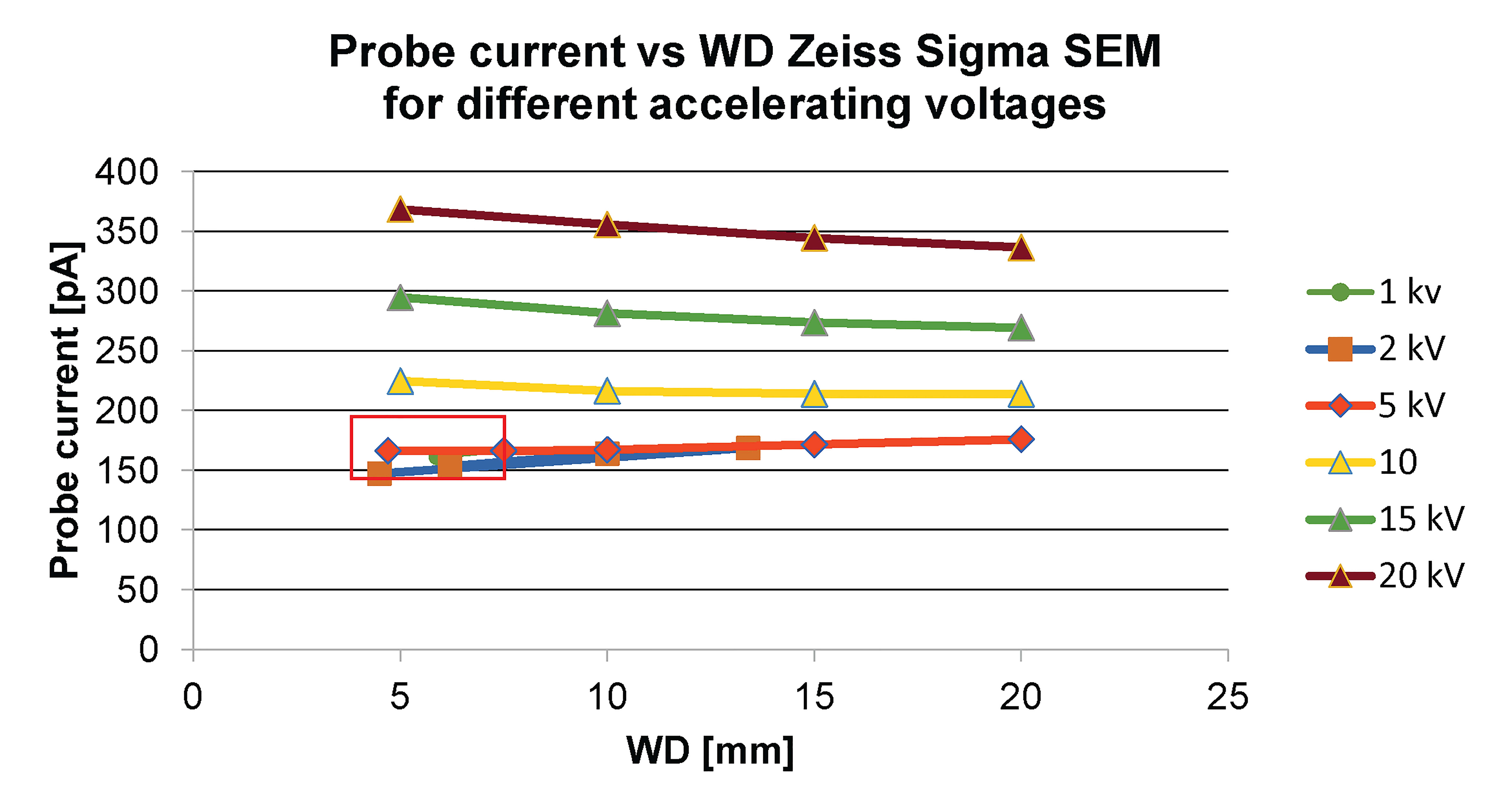

Supplement: Supplementary file 3 [file Image1.JPEG]

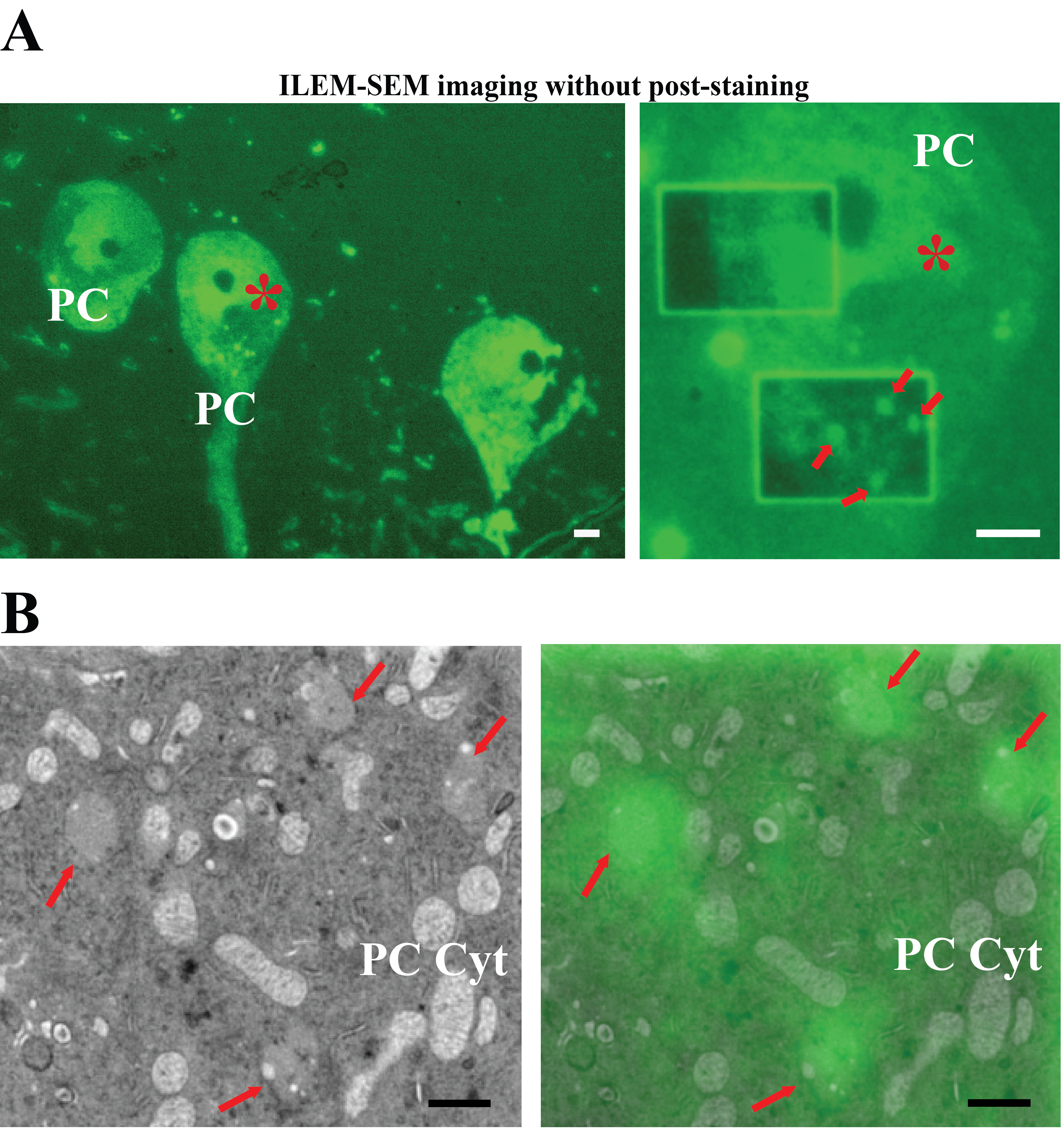

Supplement: Supplementary file 4 [file Image4.JPEG]

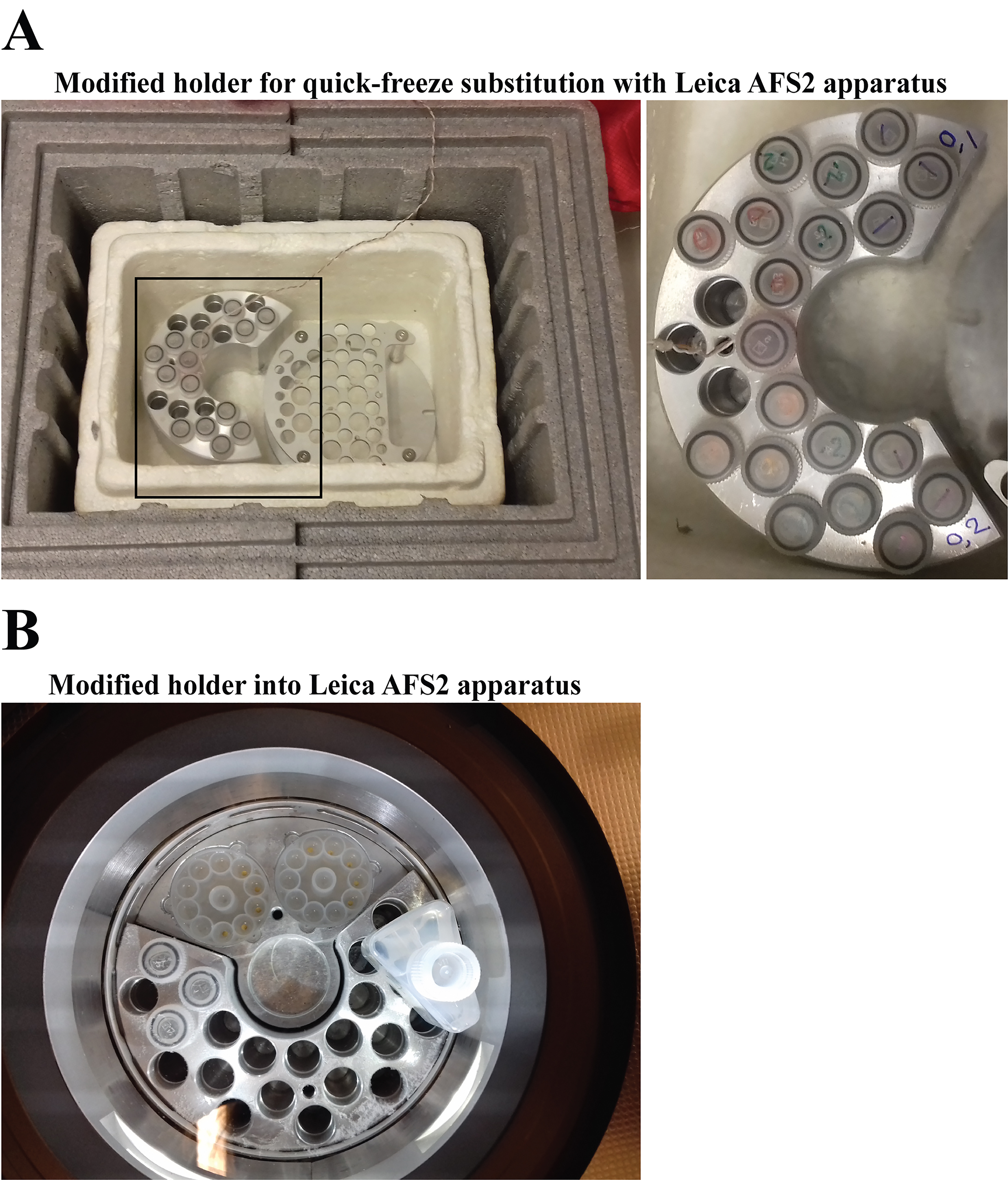

Supplement: Supplementary file 5 [file Image2.JPEG]
