# Supplementary material for: Preservation of Fluorescence Signal and Imaging Optimization for Integrated Light and Electron Microscopy
Source: Front Cell Dev Biol. 2021 Dec 15;9:737621. doi: 10.3389/fcell.2021.737621 (PMC8715528; doi:10.3389/fcell.2021.737621)
Supplement: Supplementary file 6 [file DataSheet1.docx]

Supplementary Material

**Supplementary Figure 1.** Graph showing how probe current (vertical axis) in the Zeiss Sigma was affected by WD (horizontal axis) and accelerating voltage. The red box indicates the range at which the experiments in this paper were conducted. pA: picoAmpere, WD: working distance.

**Supplementary Figure 2.** Quick-freeze substitution (QFS) holder with cryotubes containing the samples in a styrofoam box for quick freeze-substitution to -50°C **(A,** left**)** and a magnification of the boxed area **(A,** right**). (B)** The QFS-holder is fitted snugly in the space next to the metal part holding the flow rings of the Leica AFS2 freeze-substitution apparatus, to facilitate transfer of samples from the cryotubes to the flow rings.

**Supplementary Figure 3**. Gallery of cells freeze-substituted with 0.2% UrAc imaged by SEM and BSE-detector demonstrating the effects of varying acceleration voltage (in vertical direction) and section thickness (in horizontal direction) on image quality. At increasing acceleration voltage, image quality breaks down at increasing section thickness, as indicated by the red line separating the good quality from the bad quality pictures. BS-detector, dwell time 50 µs, WD 4-5 mm, except for 1kV WD = 2.5 mm. Abbreviations: BSE, backscattered electrons (Gatan OnPoint detector); SEM, scanning electron microscope; UrAc, uranyl acetate; WD, working distance. Scale bars: 1 = µm.

**Supplementary Figure 4**. More detailed observation at high magnification on the same Purkinje cells (asterisk) as displayed in Fig. 8. The bleached square marked by EM-acquisition that can be seen in the magnified part (**A** right, asterisk) of the soma of the middle Purkinje cell (**A** left, asterisk), shows 4 brighter spots (**A** right, red arrows). The corresponding ILEM-images (shown in overlay in **B** right image) show that these correspond with lysosome-like structures (**B**, red arrows), that can be better observed in the individual SEM-image (**B** left, red arrows). Apparently, the GFP-tagged calbindin occurs in higher concentrations in these organelles. Abbreviations: Cyt, cytoplasm; ILEM-SEM, integrated light and electron microscope; PC, Purkinje cell. Scale bars: top 2 = µm, bottom 0.5 = µm.

**Supplementary File MC Simulations.**

Thits file contains Monte Carlo Simulations of the interaction volumes of the electron beam at different energies (2, 5 and 10 kV) in material consisting of Carbon with different proportions of Uranium (0, 1, 10 and 100%). The interaction volumes are all very similar except for the interaction with 100% Uranium, where it is about 5 times smaller.
